# Comparative Study on Corrosion Protection of Reinforcing Steel by Using Amino Alcohol and Lithium Nitrite Inhibitors

**DOI:** 10.3390/ma8010251

**Published:** 2015-01-14

**Authors:** Han-Seung Lee, Hwa-Sung Ryu, Won-Jun Park, Mohamed A. Ismail

**Affiliations:** 1 Department of Architectural Engineering, Hanyang University, 1271 Sa 3-dong, Sangrok-gu, Ansan 426-791, Korea; E-Mails: ercleehs@hanyang.ac.kr (H.-S.L.); mismail@hanyang.ac.kr (M.A.I.); 2 Sustainable Building Research Center, Hanyang University, 1271 Sa 3-dong, Sangrok-gu, Ansan 426-791, Korea; E-Mail: jooney1010@hanyang.ac.kr

**Keywords:** reinforced concrete, reinforcing steel corrosion, amino alcohol inhibitor, electrochemical evaluation

## Abstract

In this study, the ability of lithium nitrite and amino alcohol inhibitors to provide corrosion protection to reinforcing steel was investigated. Two types of specimens—reinforcing steel and a reinforced concrete prism that were exposed to chloride ion levels resembling the chloride attack environment—were prepared. An autoclave accelerated corrosion test was then conducted. The variables tested included the chloride-ion concentration and molar ratios of anti-corrosion ingredients in a CaOH_2_-saturated aqueous solution that simulated a cement-pore solution. A concentration of 25% was used for the lithium nitrite inhibitor LiNO_2_, and an 80% solution of dimethyl ethanolamine ((CH_3_)_2_NCH_2_CH_2_OH, hereinafter DMEA) was used for the amino alcohol inhibitor. The test results indicated that the lithium nitrite inhibitor displayed anti-corrosion properties at a molar ratio of inhibitor of ≥0.6; the amino alcohol inhibitor also displayed anti-corrosion properties at molar ratios of inhibitor greater than approximately 0.3.

## 1. Introduction

Corrosion of reinforcing steel in concrete arising from the penetration of chlorides, which can result from the exposure of concrete to marine environments or ocean sand, represents one of the major factors that cause deterioration of reinforced concrete structures [1]. Reinforcing steel in reinforced concrete structures is protected by an alkali-oxide film formed during the cement hydration process [2]. However, if the chloride concentration at the reinforcing steel surface reaches a critical value, the film on the passive-state metal is destroyed, which triggers corrosion of the reinforcing steel. Chlorides in concrete come from seawater, the salinity of airborne chlorides, or de-icing salts. The infiltration of chlorides inside the concrete is controlled by the concrete’s characteristics, such as the unit amount of cement, the ratio of water to binding material, and the type of binding material used [3]. As a method of inhibiting reinforcing steel corrosion inside concrete from chlorides, the use of corrosion inhibitors during concrete preparation is becoming common practice. However, the anti-corrosion effects and proper amount of corrosion inhibitors used to inhibit reinforcing steel corrosion in reinforced concrete structures exposed to a chloride environment need to be examined. Quantitative data on the anti-corrosion effects, according to the type of corrosion inhibitor used, are difficult to obtain. Moreover, since corrosion inhibitors experience a dramatic decline in performance at high temperatures and pressures, depending on the type, new methods for evaluating the anti-corrosion properties of these inhibitors and further studies are desperately needed [4].

Reinforcing steel corrosion by chlorides acts as a major deterioration factor at the interface between reinforcing steel and concrete inside the concrete structure. The reinforcing steel corrosion is initiated once the chloride concentration exceeds a certain critical value, and it is a well-known fact that chloride ions are involved in the process of reinforcing steel corrosion [5]. There have been many studies on the critical chloride concentration. A precedent study indicated that in case the concentration ratio of [Cl^−^:OH^−^] exceeds 0.6, damage to the film on the passive-state metal occurs, initiating local corrosion [6].

Generally, corrosion inhibitors are classified as anodic or mixed reactants, depending on their reaction mechanism. Anodic corrosion inhibitors, which are mostly nitrite-based, display corrosion protection through a partial interface process. Oxidation of ferric ions forms a ferric-oxide film around the reinforcing steel. Nitrite-based inhibitors are considered the most effective products on the market. Concerns are with their toxicity, solubility, and possible increase of corrosion rate in the case of low dosage or in the presence of cracks, and also the relatively high costs of this type of additive [7,8,9,10]. Thus, the use of amino alcohol-based mixed corrosion inhibitors has increased recently, but studies related to these types of inhibitors are lacking.

A pilot study was initiated to evaluate the anti-corrosion properties of these two corrosion inhibitors, qualitatively. Tests were conducted on lithium nitrite and amino alcohol inhibitors by means of electrochemical techniques. An autoclave accelerated corrosion test that simulates accelerated reinforcing steel corrosion inside concrete was conducted [11,12,13]. The anti-corrosion effects were evaluated by measuring the speed of the reinforcing steel corrosion using a Tafel plot. The investigated variables included the chloride ion concentration and the molar ratio of inhibitor to chloride in a CaOH_2_-saturated aqueous solution that simulated a cement-pore solution. The study was intended to confirm the anti-corrosion properties of these corrosion inhibitors in relation to the chloride-ion content inside concrete [14]. The electrochemical evaluation methods used to access reinforcing steel corrosion are receiving much attention as they represent non-destructive techniques of relatively high accuracy and offer the possibility to measure small amounts of corrosion within a short period of time [15,16]. Among them, the techniques most frequently used are half-cell potential mapping, concrete resistivity, liner polarization resistance, and A/C impedance [17,18].

It is planned after confirming the outcomes of this study to launch a comprehensive program to study the anti-corrosion effects of these inhibitors on different specimens under different environments on a long-term basis.

## 2. Mechanisms of Lithium Nitrite and Amino Alcohol Inhibitors

### 2.1. Lithium Nitrite Inhibition Mechanism

**Figure 1:**
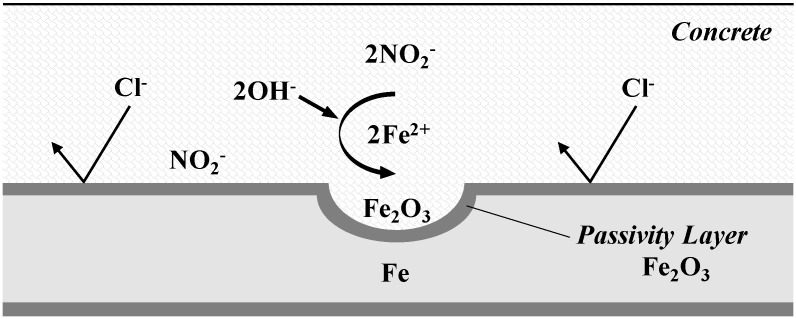
Anti-corrosive mechanism of nitrite-based corrosion inhibitors.

Reinforcing steel corrosion in a reinforced concrete structure is prevented by the formation of a film on passive state metals on the reinforcing steel surface by the high alkalinity of the concrete. This film on passive state metals (Fe_2_O_3_) is stable, with strong bonding in an alkaline environment, but it becomes unstable and dissolves in the presence of chloride ions. Accordingly, the anti-corrosion mechanism of the lithium nitrite inhibitor commonly used in reinforced concrete structures involves nitrite ions (NO_2_^−^) from nitrite reacting with ferrous ions (Fe^2+^), which inhibits the movement of Fe^2+^ from the anode, whereby Fe_2_O_3_ is deposited on the iron surface to form a film on passive state metals. As a result, the corrosion reaction is inhibited. The reaction follows Equation (1) and the mechanism is shown in Figure 1 [19].

(1) $$2{\text{Fe}}^{2+}+2{\text{OH}}^{-}+2{{\text{NO}}_{2}}^{-}\to 2{\text{NO}}_{2}↑+{\text{Fe}}_{2}O_{3}+H_{2}O$$

### 2.2. Amino Alcohol Inhibitor Mechanism

Amino alcohol corrosion inhibitors control corrosion by attacking the cathodic activity, blocking sites where oxygen picks up electrons and is reduced to hydroxyl ion. Also, inhibition of corrosion occurs through a mechanism whereby amino alcohols displace chloride ions and form a durable passivating film. In this view, although the amino alcohols adsorb on non-corroding sites, which may seem more cathodic than anodic, they can just as easily be said to adsorb on potentially anodic sites as well [20]. Ormellese *et al.* [21] suggested that organic corrosion inhibitors reduce the ingress of chloride by filling concrete pores and blocking the porosity of concrete by the formation of complex compounds. Thus, the value of chlorides reaching to the steel surface is significantly less so corrosion is inhibited. Several studies of the corrosion inhibition effect of amino alcohols on steel report their performance as a function of concentration and pH in saline solutions [22,23,24].

## 3. Testing Anti-Corrosion Characteristics of Corrosion Inhibitors

### 3.1. Test Summary

The present study assessed the electrochemical characteristics of corrosion inhibitors in aqueous solution based on the inhibitor amounts added in order to perform a qualitative evaluation of their anti-corrosion effects. First, in terms of the electrochemical anti-corrosion properties, Table 1 lists the physical properties of the tested corrosion inhibitors, and the chemical composition of the reinforcing steel is shown in Table 2.

The potentiodynamic polarization curve, known as a Tafel plot, depicts the relationship between potential and corrosion current density. This plot exhibits a linear region, the slope of which is known as the Tafel constants (anodic and cathodic Tafel constants). The intersection of the projection of the linear region of the plot with the open circuit potential (*E*_corr_) gives the cathodic or anodic corrosion current (*i*_corr_). Once *i*_corr_ is determined, the following equation, derived from Faraday’s law, can be used to calculate the corrosion rate [25]:

(2) $$\mathrm{Corrosion rate}\left(\mu m/y\right)=\;\frac{3.27\times I_{\mathrm{corr}}\times E.W.}{d}$$

The corrosion rate in Equation (2) is expressed in micrometers per year, μm/y. *I*_corr_ is the corrosion current density in µA·cm^−2^, obtained by dividing *i*_corr_ with the exposed surface area of the measured specimen. *E.W.* is the equivalent weight of steel in g, and *d* is the density of steel in g/cm^3^.

The polarization resistance *R*_p_ (Δ*E*/Δ*I*), which is the slope of the potential-current curve at *E*_corr_, is related to *I*_corr_ through the following Stern–Geary relationship [26]:

(3) $$I_{\mathrm{corr}}\left(\mu A/{\mathrm{cm}}^{2}\right)=\;\frac{\left[\beta_{a}\times \;\beta_{c}\right]}{2.3\;(\beta_{a}+\;\beta_{c})}\;R_{p}$$

β*_a_* and β*_c_* are the anodic and the cathodic Tafel constants, respectively, expressed in mV/decade of the current. *R*_p_ is expressed in KΩ·cm^2^. It is seen here that for the determination of *I*_corr_ in this technique, β*_a_* and β*_c_* are determined from the Tafel plot.

A potentiostat was used to measure the corrosion potential (*E*_corr_), corrosion current density (*I*_corr_), and corrosion rate (*CR*). Also, the pore solution was prepared by adding NaCl to saturated calcium hydroxide (solubility of 0.173 g/100 mL at 20 °C). Concentrations of chloride ions (NaCl amount added) were set to 0.6 kg/m^3^, 1.2 kg/m^3^, 2.4 kg/m^3^, and 4.8 kg/m^3^, with the chloride-ion content used as standard for the prediction of the service life set to 1.2 kg/m^3^.

The types of corrosion inhibitors used were lithium nitrite (LiNO_2_) and DMEA ((CH_3_)_2_NCH_2_CH_2_OH). The molar ratios of the anti-corrosion ingredients, based on the ratio of chloride to hydroxide ions, were set to 0.0, 0.3, 0.5, and 1.2 for the experiments. The lithium nitrite inhibitor used was a 25% solution of LiNO_2_ and the amino alcohol inhibitor used was an 80% concentrated solution. The amounts to be added were calculated and the tests were performed accordingly. Table 3 shows the different experimental parameters and their values. With respect to the chloride ion concentrations of 1.2 kg/m^3^ and 2.4 kg/m^3^, the chloride ion-dependent molar ratios of inhibitor to chloride were set to 0.0, 0.3, 0.6, and 1.2.

**Table 1:** Physical properties of inhibitors.

Type	Main Component of Inhibitor	Specific Gravity	pH	Viscosity (cps)	Solid Content (%)
Lithium Nitrite inhibitor	LiNO_2_	1.12	11.5	13	25
Amino Alcohol inhibitor	(CH_3_)_2_NCH_2_CH_2_OH	1.07	11.9	11	80

**Table 2:** Chemical composition of reinforcing steel (%).

C	Si	Mn	P	S	Ni	Cr	Mo	Cu	Sn
0.24	0.26	0.95	0.016	0.008	0.03	0.04	0.01	0.02	0.0005

**Table 3:** Experimental testing parameters conditions.

No.	Content of Cl^−^ (kg/m^3^)	Lithium Nitrite Inhibitor		Amino Alcohol Inhibitor	
		LiNO_2_		(CH_3_)_2_NCH_2_CH_2_OH	
		Molar Ratio	Addition	Molar Ratio	Addition
		[Cl^−^]/[NO_2_^−^]	kg/m^3^	[Cl^−^]/[OH^−^]	kg/m^3^
1	0.0	0.0	0.00	0.0	0.00
2	0.6	0.0	0.00	0.0	0.00
3		0.3	1.08	0.3	0.45
4		0.6	2.15	0.6	0.91
5		1.2	4.30	1.2	1.81
6	1.2	0	0.00	0.0	0.00
7		0.3	2.15	0.3	0.91
8		0.6	4.30	0.6	1.81
9		1.2	8.60	1.2	3.62
10	2.4	0.0	0.00	0.0	0.00
11		0.3	4.30	0.3	1.81
12		0.6	8.60	0.6	3.62
13		1.2	17.21	1.2	7.24
14	4.8	0.0	0.00	0.0	0.00
15		0.3	8.60	0.3	3.62
16		0.6	17.21	0.6	7.24
17		1.2	34.41	1.2	14.48

Tests were carried out first on reinforcing steel specimens in solutions that contain different inhibitor and NaCl concentrations to determine the best molar ratio for the inhibitor before the second stage was carried out. The second stage involved studying the effect of the best inhibitor molar ratio calculated from stage one on the anti-corrosion protection of reinforcing steel in concrete that was subjected to chloride attack.

To suggest an effective measure for reinforcing steel corrosion inhibition inside concrete, the reinforcing steel corrosion conditions were examined after corrosion acceleration of the reinforced concrete had taken place. The corrosion acceleration of the reinforcing steel in concrete was done using an autoclave. The autoclave method is an accelerated corrosion method that conforms to Korean standard KS F 2599-1 [11].

For a reinforced concrete specimen, the corrosion potentials were measured by using the half-cell potential technique, and the corrosion-area ratio was calculated by confirming the reinforcing steel corrosion conditions after the application of the accelerated corrosion method for reinforced concrete. The amount of water-soluble chloride, which directly affects reinforcing steel corrosion, was measured using a potentiometric titration apparatus, in accordance with ASTM C 1218 [27]. Then, the results were analyzed. Table 4 shows the composition and the physical properties of the cement and Table 5 shows the physical properties of the aggregates. The mix proportion of the concrete is indicated in Table 6. The tests were conducted using coarse aggregate with a nominal maximum size of 25 mm, a water to cement ratio of 0.60, and a unit cement amount of 300 kg/m^3^. The size of the test specimens was 40 × 40 × 160 mm^3^.

**Table 4:** Chemical composition and physical properties of cement. LOI: Loss on ignition.

Chemical Composition (%)							Specific Surface (cm^2^/g)
SiO_2_	Al_2_O_3_	Fe_2_O_3_	CaO	MgO	SO_3_	LOI	
21.95	6.59	2.81	60.12	3.32	2.11	2.58	3.14

**Table 5:** Physical properties of aggregates.

Type	Density (kg/m^3^)	Absorption (%)	Fineness Modulus
Fine aggregate	2.58	1.34	2.57
Coarse aggregate	2.70	1.4	6.83

**Table 6:** Mix proportion of concrete. W/C: water–cement ratio; S/a: fine aggregate percentage.

W/C (%)	Air (%)	S/a (%)	Weight Mixing (kg/m^3^)				
			Water (kg/m^3^)	Cement (kg/m^3^)	Fine Aggregates (kg/m^3^)	Coarse Aggregates (kg/m^3^)	Admixture (C × %)
60	4.5	43	186	300	836	1033	0.5

### 3.2. Electrochemical Testing of Anti-Corrosion Properties

**Figure 2:**
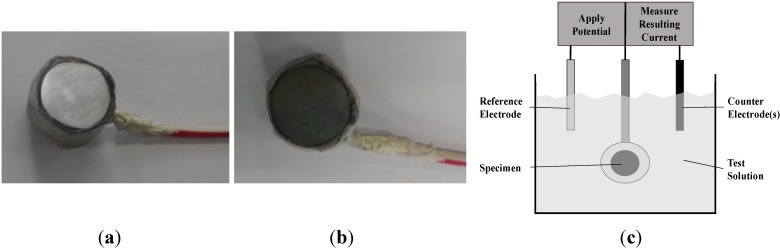
Reinforcing steel specimen and schematic diagram of the test rig. (**a**) Specimen (Before); (**b**) Specimen (After); (**c**) Schematic diagram of the potentiostat.

**Figure 3:**
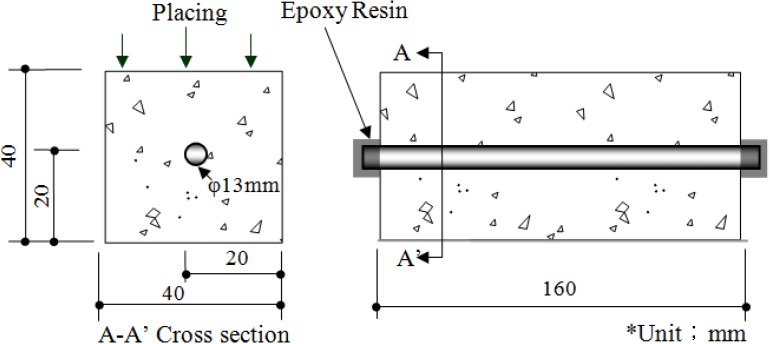
Details of test specimen.

To measure the corrosion status of reinforcing steel in NaCl solution, a circular reinforcing steel section with a diameter of 10 mm was cut to a length of 10 mm and a wire was welded on one side to provide an electrical contact. After that, the entire specimen was insulated with a silicone coating except for the areas needed for the measurement of the electrochemical characteristics. The prepared specimen was soaked in CaOH_2_ solution for 10 min in order to reach a stable state before testing. The electrochemical properties, such as the corrosion potential and corrosion rate, were then measured. Figure 2 shows photos of the reinforcing steel specimen before and after the accelerated corrosion procedure and a schematic diagram of the test rig used in the experiments. Figure 3 shows the details of the reinforced concrete specimen.

#### 3.2.1. Interpretation of Tafel Plot Curves

Qualitative determination of the level of corrosion protection of the reinforcing steel can be done by calculating the corrosion current density as in Equation (2). The corrosion rate then can be calculated as in Equation (1). In the experiments, 10 mV/min was used as the potential sweep rate for the corrosion current density measurements and the potential-difference range was set to 0–1000 mV.

#### 3.2.2. Experiments on Reinforcing Steel Anti-Corrosion Properties by the Accelerated-Corrosion Method

**Figure 4:**
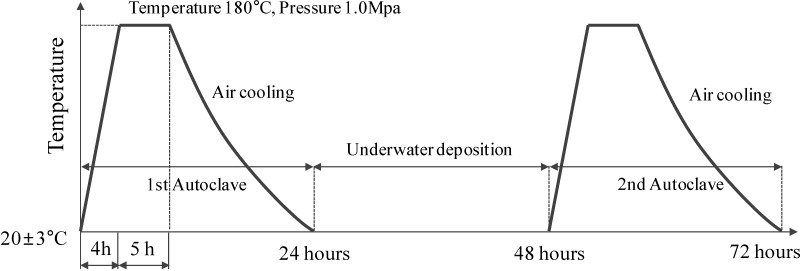
Temperature profile of autoclave-accelerated corrosion.

**Figure 5:**
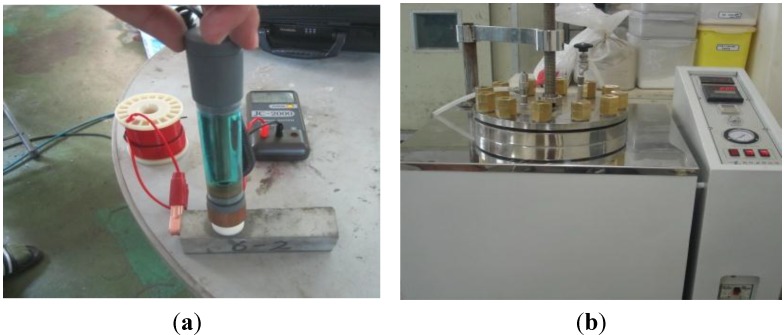
Procedures for testing the anti-corrosion characteristics. (**a**) Measurement of the corrosion potential; (**b**) Accelerated corrosion by autoclave.

As shown in Figure 4, the corrosion of a reinforced concrete specimen was accelerated by the autoclave accelerated method, in accordance with autoclave accelerated corrosion test [11]. Figure 5 shows the procedure for measuring the reinforcing steel anti-corrosion properties with: “Figure 5a” measuring the corrosion potential; “Figure 5b” the autoclave-accelerated corrosion test, and then the determination of the soluble chloride content using the potentiometric titration apparatus.

After the completion of the accelerated corrosion tests, the corrosion potentials were measured by the half-cell potential technique, as shown in Figure 5, and the corrosion/area ratio was estimated by scanning the corrosion shape with a transparent sheet and using automated area-measurement software.

For measuring the water-soluble chloride content, which has a direct impact on reinforcing steel corrosion, a potentiometric titration apparatus was used in accordance with the ASTM C 1218 guidelines [27]. For the measurements of the water-soluble chloride content after accelerated corrosion, the test specimen was split using a universal testing machine, and a 10 g powder sample at a distance of 10 mm from the surface of the concrete was collected, again in accordance with ASTM C 1218 [27]. The 10-g powder sample was well mixed in a beaker with 50 mL of ion-exchange solution. Next, approximately 3 mL of hydrogen peroxide (H_2_O_2_) was added to eliminate any effects of sulfides. To prevent any changes in the chloride concentration originating from evaporation of the sample filtrate, heating for 5 min took place with a watch glass cover. The sample was then kept under atmospheric conditions for 24 h, after which the chloride extract was filtered with a filtering assembly. The chloride content was measured using a potentiometric titration apparatus and the water-soluble chloride content in the specimen was quantified.

## 4. Test Results

### 4.1. Electrochemical Anti-Corrosion Characteristics of Corrosion Inhibitors

The test results are listed in Table 7. It is known from previous studies that corrosion is initiated if the molar ratio exceeds the value of 0.6, regardless of the type of corrosion inhibitor. However, the results of this study indicated that the lithium nitrite inhibitor most strongly exhibited corrosion-protection properties at a molar ratio of 0.6 and above, whereas the amino alcohol inhibitors exhibited corrosion protection properties starting at a molar ratio of 0.3. From these results, it was determined that the anti-corrosion properties of an amino alcohol inhibitor were superior to those of a lithium nitrite inhibitor at the same molar ratio.

**Table 7:** Electrochemical measurement results of reinforcing steel specimen in solution. CSE: copper–copper sulfate electrode.

No.	Content of Cl^−^ (kg/m^3^)	Lithium Nitrite Inhibitor				Amino Alcohol Inhibitor			
		LiNO_2_				(CH_3_)_2_NCH_2_CH_2_OH			
		*E*_corr_	*I*_corr_	*CR*	*R*_p_	*E*_corr_	*I*_corr_	*CR*	*R*_p_
		mV. CSE	μA/cm^2^	mpy	Ω cm^2^	mV. CSE	μA/cm^2^	mpy	Ω cm^2^
1	0.0	−207	0.092	0.20	282.6	−207	0.092	0.20	282.6
2	0.6	−359	0.98	1.22	26.53	−359	0.98	1.22	38.81
3		−350	0.73	0.98	35.67	−312	0.22	0.28	100.00
4		−333	0.46	0.77	39.16	−309	0.21	0.22	118.18
5		−283	0.19	0.44	63.41	−208	0.11	0.13	166.40
6	1.2	−381	1.19	1.33	21.85	−381	1.19	1.33	21.85
7		−358	0.87	1.02	29.89	−298	0.20	0.35	106.56
8		−311	0.55	0.85	47.27	−291	0.19	0.24	122.64
9		−318	0.20	0.32	81.25	−290	0.18	0.14	144.44
10	2.4	−430	1.26	1.55	20.63	−430	1.26	1.55	20.63
11		−368	1.05	1.45	24.76	−324	0.21	0.27	83.60
12		−340	0.45	0.88	57.78	−278	0.11	0.22	146.09
13		−338	0.22	0.62	86.67	−262	0.10	0.14	170.50
14	4.8	−438	1.50	1.76	17.33	−438	1.5	1.76	17.33
15		−388	0.90	1.54	28.89	−360	0.33	0.19	60.19
16		−344	0.86	0.94	30.23	−311	0.19	0.17	95.94
17		−327	0.23	0.43	49.06	−302	0.18	0.14	123.81

#### 4.1.1. Corrosion Potential for Different Types of Corrosion Inhibitors

**Figure 6:**
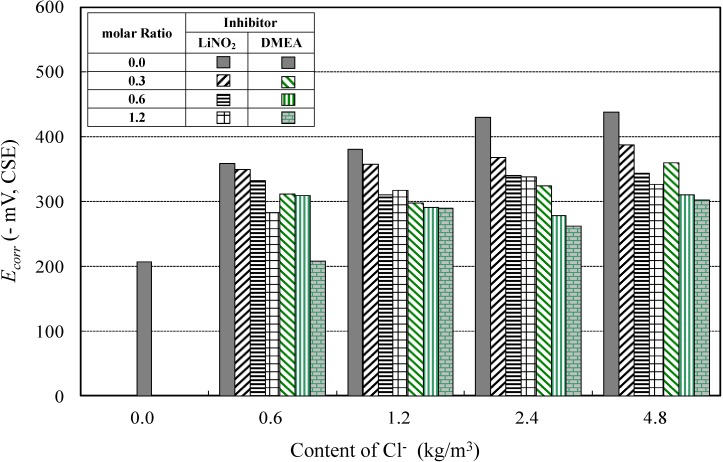
Corrosion potential of the different inhibitors.

Figure 6 shows the relationship between the corrosion potentials at different molar ratios of inhibitor and chloride concentrations for the lithium nitrite and amino alcohol inhibitors. One can see from Figure 6 that in the case of no inhibitor, the corrosion potential increases as the content of Cl^−^ increases and once the inhibitor is added, the corrosion potential decreases. It is also evident that at the same content of Cl^−^ and similar molar ratio in both inhibitors, the corrosion potential achieved with DMEA is lower than that of lithium nitrite. This observation is noticed at all concentrations of Cl^−^.

#### 4.1.2. Corrosion Current Density for Different Types of Corrosion Inhibitors

**Figure 7:**
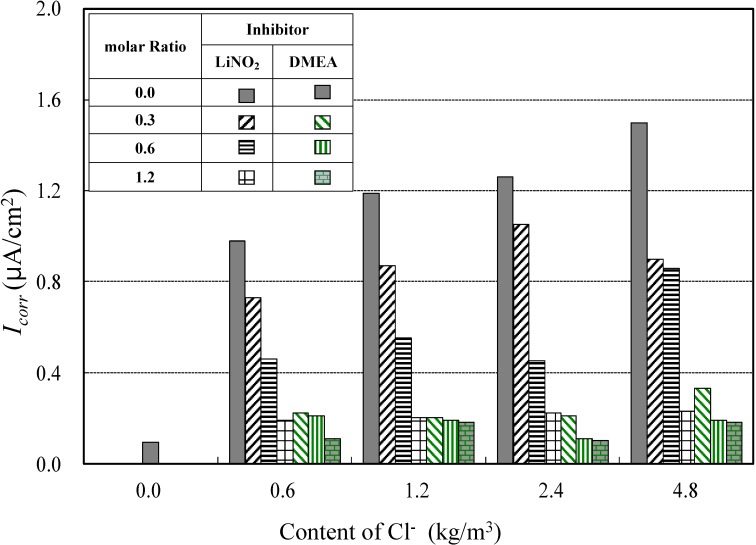
Corrosion current density of the different inhibitors.

Figure 7 shows the relationship between corrosion current density values at different molar ratios of lithium nitrite and amino alcohol inhibitors and concentration of Cl^−^. The corrosion current densities were measured at the initiation of reinforcing steel corrosion. Under the presumption that the corrosion rate is considered low when corrosion current density value is in the range 0.2–0.5 μA/cm^2^ [28], both the lithium nitrite and amino alcohol inhibitors showed decreasing corrosion current densities as the molar ratio of the inhibitor increases. Moreover, as is evident from Figure 7, if the corrosion status is evaluated on the basis of the corrosion current density, in the case of the lithium nitrite inhibitor, the corrosion current density was distributed in the range of 0.2–1.0 μA/cm^2^, depending on the chloride ion content and the molar ratio of the inhibitor. Anti-corrosion properties (corrosion rate is passive) are present at chloride ion levels of 0.6 kg/m^3^ and 1.2 kg/m^3^ and at the molar ratio of inhibitor of 1.2. At chloride ion levels of 2.4 kg/m^3^ and 4.8 kg/m^3^, corrosion was low at molar ratios of inhibitor of ≥1.2.

On the other hand, for the amino alcohol inhibitor with a corrosion current density of up to 0.2 μA/cm^2^, anti-corrosion properties (corrosion rate is passive) are present at chloride ion levels of 0.6 kg/m^3^ and 1.2 kg/m^3^ and at the molar ratio of inhibitor of 1.2. At chloride ion levels of 2.4 kg/m^3^ and 4.8 kg/m^3^, corrosion was low at molar ratios of inhibitor to chloride of ≥0.6, whereupon anti-corrosion properties are clearly evident.

#### 4.1.3. Corrosion Rates for the Different Types of Corrosion Inhibitors

**Figure 8:**
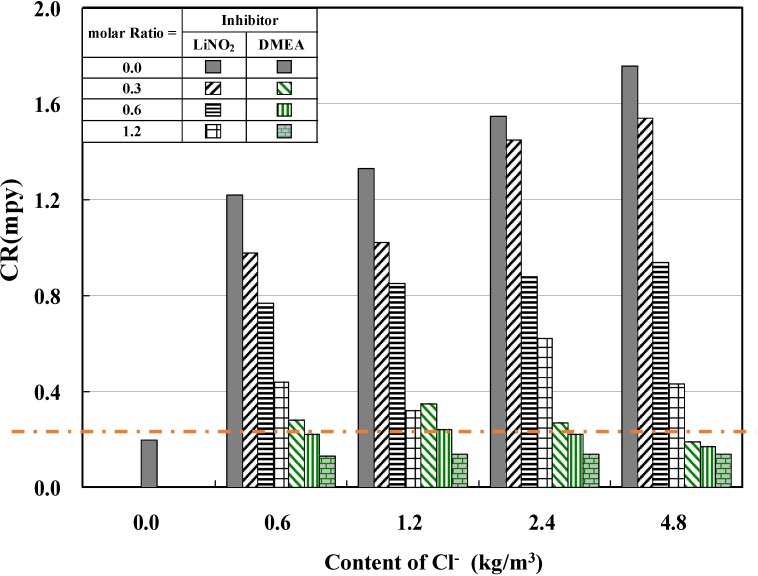
Relationship between corrosion rate and content of Cl^−^.

Figure 8 shows the relationship between corrosion rate and concentration of Cl^−^ for lithium nitrite and amino alcohol inhibitors at different molar ratios of inhibitor, in which the anti-corrosion capability was evaluated; it should be below the anti-corrosion standard of 0.5 mpy (dashed horizontal line in Figure 8). The lithium nitrite inhibitor, LiNO_2_, showed a decrease in corrosion rate with increasing inhibitor molar ratio. In particular, only if the inhibitor molar ratio was ≥1.2, the anti-corrosion standard of 0.5 mpy was satisfied. Likewise, the corrosion rate of the amino alcohol inhibitor tended to decrease as the inhibitor molar ratio increased. In particular, the corrosion rate of DMEA quickly dropped below 0.5 mpy in all cases in which the inhibitor molar ratio was ≥0.3, regardless of chloride ion levels of 0.6 kg/m^3^, 1.2 kg/m^3^, 2.4 kg/m^3^, and 4.8 kg/m^3^. These results indicate that a small amount of amino alcohol corrosion inhibitor can achieve outstanding anti-corrosion capabilities.

Therefore, from the analysis results of both corrosion current density and corrosion rate, the lithium nitrite inhibitor achieved anti-corrosion properties at a molar ratio of approximately 1.2 or greater, whereas the amino alcohol inhibitor achieved anti-corrosion properties at a molar ratio above 0.3. Considering that all results were obtained in an aqueous solution containing chloride ions, even small amounts of amino alcohol inhibitor show better anti-corrosion capabilities than the lithium nitrite inhibitor.

#### 4.1.4. Polarization Resistances for the Different Types of Corrosion Inhibitors

**Figure 9:**
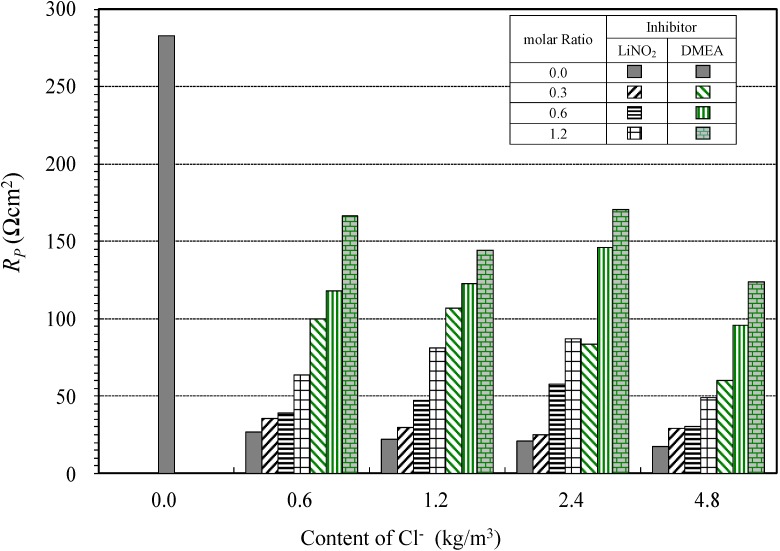
Relationship between polarization resistance and molar ratio.

Figure 9 shows the relationship between the polarization resistance and content of Cl^−^ at different molar ratios of lithium nitrite and amino alcohol inhibitors. The polarization resistance of the lithium nitrite inhibitor, LiNO_2_, tended to increase with an increase in the molar ratio of the lithium nitrite inhibitor.

Thus, it is established that polarization resistance is not an appropriate indicator of anti-corrosion properties. The reason for this is believed to be the nonlinearity of the slopes of the anodic and cathodic polarization curves, which indicates that varying values of the corrosion current density *I*_corr_ may appear, depending on the detailed test conditions. On the other hand, the polarization resistance of the amino alcohol inhibitors also tended to increase with an increasing molar ratio of inhibitor to chloride.

### 4.2. Testing of Anti-Corrosion Properties of Reinforced Concrete Specimen by the Accelerated Corrosion Method

**Figure 10:**
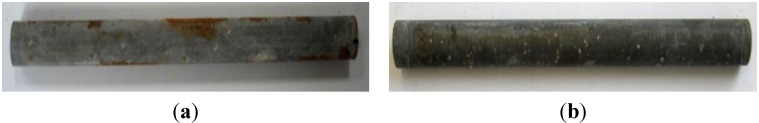
Appearance of reinforcing steel corrosion at the chloride ion concentration of 2.4 kg/m^3^ and inhibitor molar ratio of 1.2. (**a**) Lithium nitrite inhibitor (LiNO_2_); (**b**) Amino-alcohol inhibitor (DMEA).

Table 8 shows the experimental test results of the reinforced concrete specimen that were subjected to accelerated corrosion tests. Figure 10 shows the corrosion in reinforcing steel after being taken from the reinforced concrete specimen. A reinforced concrete specimen was subjected to a chloride ion content of 2.4 kg/m^3^ and a molar ratio of inhibitor of 1.2. Table 9 displays the corrosion conditions of the reinforcing steel after accelerated corrosion relative to the amount of corrosion inhibitor added. As can be seen from the test results in Table 8, as the molar ratio of the inhibitor increased—in other words, as the amount of added corrosion inhibitor increased—the half-cell potential and the corrosion/area ratio rapidly decreased, clearly indicating the corrosion-inhibiting effects.

**Table 8:** Experimental test results of the reinforced concrete specimen. CSE: copper–copper sulfate electrode.

No.	Content of Cl^−^ (kg/m^3^)	Molar Ratio	Lithium Nitrite Inhibitor			Amino Alcohol Inhibitor		
			LiNO_2_			(CH_3_)_2_NCH_2_CH_2_OH		
			Half-Cell Potential	Corrosion Area Ratio	Water-Soluble Cl^−^	Half-Cell Potential	Corrosion Area Ratio	Water-Soluble Cl^−^
			mV. CSE	%	%	mV. CSE	%	%
1	0.0	0.0	−211	11.6	0.0037	−211	11.6	0.0037
2	1.2	0.0	−387	68.5	0.0148	−387	68.5	0.0148
3		0.3	−369	34.0	0.0188	−263	13.9	0.0071
4		0.6	−343	30.6	0.0217	−213	13.1	0.0037
5		1.2	−289	18.8	0.0185	−180	6.3	0.0012
6	2.4	0.0	−443	70.6	0.0275	−443	70.6	0.0275
7		0.3	−384	34.3	0.0282	−317	21.1	0.0084
8		0.6	−352	31.5	0.0297	−229	11.5	0.0044
9		1.2	−317	13.8	0.0239	−215	8.0	0.0024

Considering the half-cell potential values and the probability of corrosion as per ASTM C 876-09 [29], the lithium nitrite inhibitor, LiNO_2_, did not achieve anti-corrosion properties as per the above mentioned standard, as the half-cell potential values were all below −350 mV; this indicates a high corrosion rate and the probability of more than 90% corrosion occurring. However, the data also show that adding lithium nitrite helped in reducing the half-cell potential values measured, compared to cases where no inhibitor was added.

On the other hand, the amino alcohol inhibitor, DMEA, showed obvious anti-corrosion properties starting from a molar ratio of inhibitor of 1.2. Also, in terms of the water-soluble chloride content, which has a direct impact on reinforcing steel corrosion, the lithium nitrite inhibitor showed a slight decrease, whereas amino alcohol inhibitors showed a drastic decrease of the chloride content with an increasing amount of the added corrosion inhibitor. These results indicate that the amino alcohol inhibitor has a superior anti-corrosion effect in a saline environment because of its chloride ion binding property.

**Table 9:** Corrosion states of the reinforcing steel after accelerated corrosion.

Content of Cl^−^	Inhibitor Molar Ratio	Corrosion Area of Rebar	
		LiNO_2_	DMEA
0.0 kg/m^3^	0.0	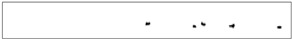	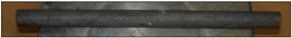
1.2 kg/m^3^	0.0	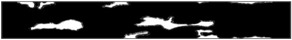	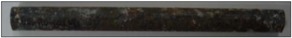
	0.3	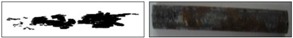	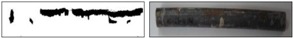
	0.6	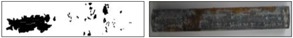	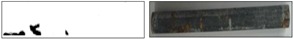
	1.2	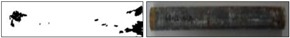	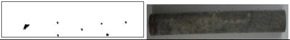
2.4 kg/m^3^	0.0	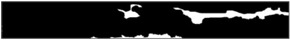	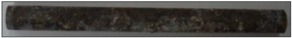
	0.3	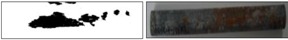	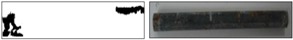
	0.6	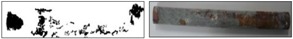	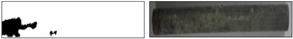
	1.2	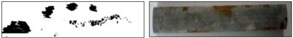	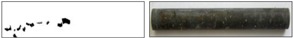

#### 4.2.1. Half-Cell Potentials of the Reinforced Concrete Specimens for Different Corrosion-Inhibitor Types

**Figure 11:**
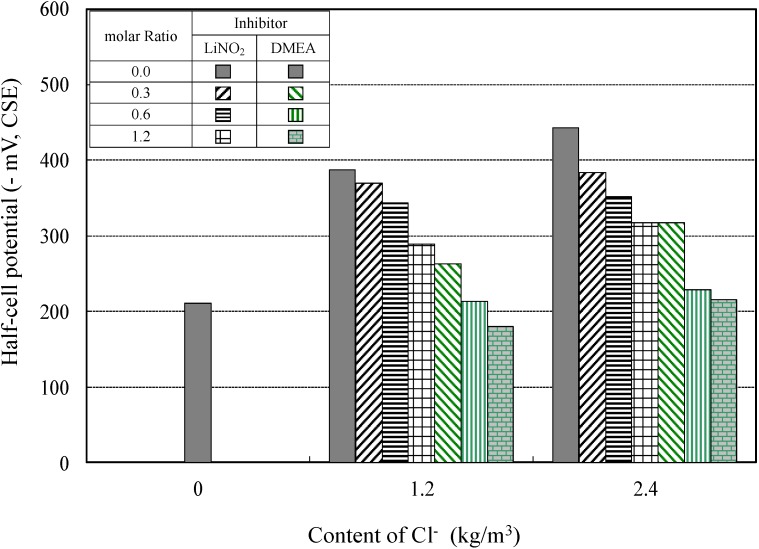
Corrosion potential of the rebar at different molar ratios of inhibitor.

Figure 11 shows the relationship between half-cell potential values and content of Cl^−^ at different molar ratios of lithium nitrite and amino alcohol inhibitors. For all investigated inhibitors, it was confirmed that as the molar ratio of the inhibitor increased, the half-cell potential negative values decreased. As indicated in Figure 11, considering only half-cell potentials of ≤−200 mV, the standard level for achieving anti-corrosion properties (ASTM C 876-09) [29], the lithium nitrite inhibitor, LiNO_2_, did not achieve anti-corrosion capabilities at any of the molar ratio used. On the other hand, the amino alcohol inhibitor immediately demonstrated anti-corrosion properties at the two chloride ion concentrations of 1.2 kg/m^3^ and 2.4 kg/m^3^ for a molar ratio of inhibitor of 1.2.

#### 4.2.2. Corrosion Areas of Reinforcing Steel in Reinforced Concrete Specimens for the Different Corrosion-Inhibitor Types

**Figure 12:**
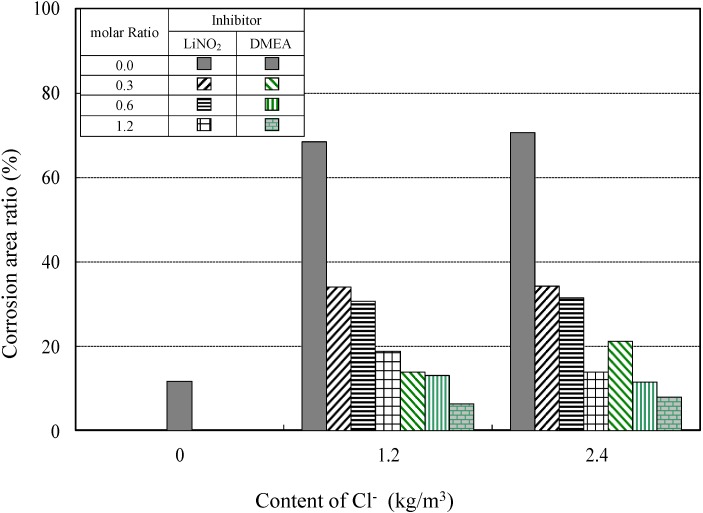
Corrosion/area ratio at various molar ratios of inhibitor.

Figure 12 displays the corrosion-area ratios related to the molar ratio of lithium nitrite and amino alcohol inhibitors. As evidenced by Figure 12, the amino alcohol inhibitor has superior anti-corrosion properties because the corrosion area is smaller when the amino alcohol rather than the lithium nitrite inhibitor is added.

In particular, at a chloride ion concentration of 1.2 kg/m^3^ and a molar ratio of inhibitor of 0.3, a corrosion/area ratio of 34.0% was observed in cases where the lithium nitrite inhibitor was added, in comparison to a corrosion/area ratio of 13.9% for the amino alcohol inhibitor under the same conditions, which represents a 2.5-fold decrease in the corrosion area.

Additionally, when using the amino alcohol inhibitor at chloride ion concentrations of 1.2 kg/m^3^ and 2.4 kg/m^3^, as the molar ratio of the inhibitor increased, corrosion drastically decreased, which indicates that its corrosion-prevention capabilities were much better than those of the lithium nitrite inhibitor.

#### 4.2.3. Chlorides in Hardened Concrete for Different Corrosion-Inhibitor Types

**Figure 13:**
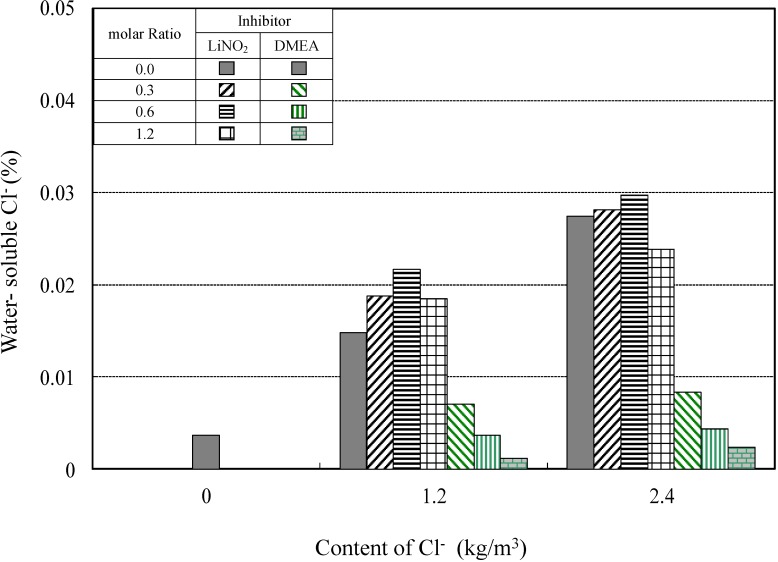
Relationship between water-soluble chloride content and content of Cl^−^ added at different molar ratios of corrosion inhibitor.

Figure 13 shows the water-soluble chloride content in hardened concrete at different molar ratios of lithium nitrite and amino alcohol inhibitors. In terms of the soluble chloride levels, which have a direct impact on reinforcing steel corrosion, the water-soluble chloride content only slightly varied as the added amount of the lithium nitrite corrosion inhibitor increased. In contrast, in cases where the amount of the amino alcohol inhibitor increased, the water-soluble chloride content showed a rapid decrease. Particularly, this rapid decrease in chloride content was accomplished already at a molar ratio of inhibitor to chloride of 0.3, where the anti-corrosion effects of the amino alcohol inhibitor in saline environments are superior to those of the lithium nitrite inhibitor due to the binding effect with respect to chloride ions.

## 5. Conclusions

Based on the present experimental results, the following conclusions are drawn:

1. Based on the half-cell potential results, the amino alcohol inhibitor was evaluated to have superior anti-corrosion capabilities compared with the lithium nitrite inhibitor.
2. The results of this study indicated that the lithium nitrite inhibitor most strongly exhibited corrosion-protection properties at a molar ratio of 0.6 and above, whereas the amino alcohol inhibitors exhibited corrosion-protection properties starting at a molar ratio of 0.3.
3. The lithium nitrite inhibitor secured anti-corrosion capacity at chloride ion levels of 2.4 kg/m^3^ and 4.8 kg/m^3^; corrosion was low at molar ratio of inhibitor of ≥1.2. Amino alcohol inhibitor DMEA showed also anti-corrosion capacity at chloride ion levels of 2.4 kg/m^3^ and 4.8 kg/m^3^; corrosion was low at molar ratios of inhibitor to chloride of ≥0.6.
4. Water-soluble chloride content only slightly varied as the amount of the lithium nitrite corrosion inhibitor increased. In contrast, in cases where the amount of the amino alcohol inhibitor increased, the water-soluble chloride content showed a rapid decrease. Particularly, this rapid decrease in chloride content was accomplished already at a molar ratio of inhibitor to chloride of 0.3.
5. Through the effect of binding chloride ions, the amino alcohol inhibitor showed anti-corrosion effects in saline environments that were clearly superior to those of the lithium nitrite inhibitor.
